# Pyrodiversity promotes interaction complementarity and population resistance

**DOI:** 10.1002/ece3.6210

**Published:** 2020-03-26

**Authors:** Lauren C. Ponisio

**Affiliations:** ^1^ Department of Entomology University of California, Riverside Riverside CA USA

**Keywords:** fire, functional complementarity, functional redundancy, mutualism, plant–pollinator network, resistance, wild bees

## Abstract

Theory predicts that network characteristics may help anticipate how populations and communities respond to extreme climatic events, but local environmental context may also influence responses to extreme events. For example, altered fire regimes in many ecosystems may significantly affect the context for how species and communities respond to changing climate. In this study, I tested whether the responses of a pollinator community to extreme drought were influenced by the surrounding diversity of fire histories (pyrodiversity) which can influence their interaction networks via changing partner availability. I found that at the community level, pyrodiverse landscapes promote functional complementarity and generalization, but did not consistently enhance functional redundancy or resistance to simulated co‐extinction cascades. Pyrodiversity instead supported flexible behaviors that enable populations to resist perturbations. Specifically, pollinators that can shift partners and network niches are better able to take advantage of the heterogeneity generated by pyrodiversity, thereby buffering pollinator populations against changes in plant abundances. These findings suggest that pyrodiversity is unlikely to improve community‐level resistance to droughts, but instead promotes population resistance and community functionality. This study provides unique evidence that resistance to extreme climatic events depends on both network properties and historical environmental context.

## Graphical abstract

As the world continues to lose species and individuals at an alarming rate, it has become increasingly imperative to understand what enables communities to maintain function in the face of the resulting shifts in composition. Using an extensive data of plant–pollinator interactions collected across a gradient of fire history diversity, or “pyrodiversity,” in Yosemite National Park, I find pyrodiversity creates the biotic infrastructure for species to change their partners and network roles—enabling species to maintain their populations following an extreme drought. Predicted shifts toward less‐diverse fire regimes will negatively influence population resistance in this and other forested ecosystems.

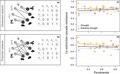

## 1 INTRODUCTION

Extreme climatic events are widely recognized as a key threat to terrestrial biodiversity worldwide (Sheffield & Wood, 2008). Droughts, in particular, are known to affect community richness and composition by causing population declines and even extinctions (Ehrlich et al., 1980; Harrison, 2000; Minckley, Roulston, & Williams, 2013; Tilman & El Haddi, 1992). More frequent and severe droughts are expected in the twenty‐first century across many regions (Alexander et al., 2009; Cook, Ault, & Smerdon, 2015; Diffenbaugh, Swain, & Touma, 2015; Stocker, 2014; Touma, Ashfaq, Nayak, Kao, & Diffenbaugh, 2015), and the resulting loss of species and their interactions will be a major driver of ecosystem change including the loss of critical ecosystem functions (Hooper et al., 2012). Understanding what enables communities to maintain function under predicted perturbations—their ability to resist, or remain “essentially unchanged” (Grimm & Wissel, 1997)—is crucial for restoration and informing conservation priorities (Oliver et al., 2015).

**FIGURE 1:**
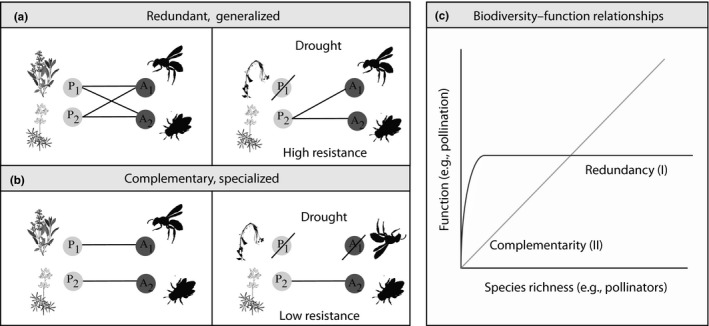
(a) Networks where interactions are redundant and generalized are more resistant to perturbations. In contrast, (b) networks where interactions are more complementary and specialized are less resistant to perturbations like the loss of species. (c) Hypothetical relationships between species richness and functional redundancy or complementarity

A central tenet of Biodiversity Ecosystem Function Theory is that species functional redundancy, whereby multiple species provide the same ecosystem function, promotes community resistance (Allan et al., 2011; Mouillot, Graham, Villéger, Mason, & Bellwood, 2013; Oliver et al., 2015). In redundant communities, if a particular species goes extinct the ecosystem functions provided by that species' interactions would still be maintained by the remaining species in the community. The loss of species in this community has no impact on overall function provision (e.g., “Biodiversity insurance hypothesis,” Lawton & Brown, 1994; Yachi & Loreau, 1999). Redundancy and generalization are related when high generalization leads to high functional niche overlap and thus redundancy. In ecological networks, because function is sustained through interspecific interactions, community resistance is highest when many species share interaction partners and are subsequently functionally redundant and generalized (Figure 1a; Lever, Nes, Scheffer, & Bascompte, 2014; Oliver et al., 2015). These communities are also predicted to be more resistant to co‐extinction cascades, where species extinction in one trophic level leads to species extinction in another, interacting trophic level (Figure 1a; Devoto, Bailey, Craze, & Memmott, 2012; Dunne, Williams, & Martinez, 2002; Lever et al., 2014; Memmott, Waser, & Price, 2004; Schleuning, Fründ, & Garca, 2015). On the other side of the interaction pattern spectrum, complementary networks occur when most species do not share partners. If species must specialize in order to partition interaction partners, complementarity and specialization will go hand‐in‐hand (Figure 1b). In communities of the same species richness, complementary networks are predicted to provide more total function than redundant networks (Figure 1c). These networks, however, are more likely to have co‐extinction cascades and failures in ecosystem function because each species contributes a unique function (Memmott et al., 2004). Although there is substantial theory and empirical work on the relationship between resistance and functional redundancy or complementarity within a single trophic level (Mouillot et al., 2013; Oliver et al., 2015), there have been no empirical tests of whether these interaction patterns relate to network resistance.

In addition to functional redundancy at the community level, individuals have the capacity to respond to perturbations through flexible behavioral strategies that promote their survival (Hofmann & Todgham, 2010; Oliver et al., 2015). For example, when species are lost due to extreme climatic events, their interaction partners may shift to the remaining species, thereby maintaining the network (Kaiser‐Bunbury, Muff, Memmott, Müller, & Caflisch, 2010; Ponisio, Gaiarsa, & Kremen, 2017). This ability to “re‐wire” interactions, or interaction flexibility, is known to increase species persistence between years (Ponisio et al., 2017) and over thousand‐year time scales (Yeakel et al., 2014). Species that cannot change their interactions patterns, such as resource specialists, may be more vulnerable to the loss of interaction partners following a disturbance (Saavedra, Stouffer, Uzzi, & Bascompte, 2011; Vidal et al., 2014).

Both functional redundancy and interaction flexibility are inherently limited by the species richness of a community. Function can reach an asymptote quickly if species are functionally redundant (Figure 1a,c), or accumulate linearly with species richness if species are perfectly complementary (Figure 1b,c II). Species richness also limits re‐wiring, because for species to re‐wire, there must be alternative species with which to interact. Local environmental heterogeneity, in turn, is a fundamental driver of the richness of communities (e.g., diversity begets diversity; Rosenzweig, 1995; Stein, Gerstner, & Kreft, 2014). Previous investigations have found that high pyrodiversity—variation in the fire history of a landscape, such as fire extent, severity, and frequency (Martin & Sapsis, 1992)—promotes landscape heterogeneity and therefore helps to promote biodiversity (Ponisio, Wilkin, et al., 2016). Spatial variation in fire history generates successional and structural habitat diversity, allowing a greater number of species to coexist across the landscape (Bowman et al., 2016; Brown & York, 2017; Burrows, 2008; Kelly & Brotons, 2017; Martin & Sapsis, 1992; Parr & Brockett, 1999; Ponisio, Wilkin, et al., 2016; Tingley et al., 2016). Depending on the accumulation of functional redundancy versus complementarity and species richness (Figure 1c I vs. II), pyrodiversity could affect ecological network resistance and total ecosystem function. If functional complementarity increases nonlinearly with species richness (Figure 1c, I), pyrodiversity will enhance plant and pollinator functional redundancy. Pyrodiversity could also increase generalization if plants/pollinators in pyrodiverse areas interact with more partners. Higher interaction redundancy and generalization would translate into increased resistance to co‐extinction cascades, particularly when species can re‐wire their interactions. In contrast, if the interaction partners of species within a community have minimal overlap, functional complementarity will be positively related to species richness and pyrodiversity. In this case, pyrodiversity could also increase generalization if species in more pyrodiverse areas interact with more species while still partitioning interactions, or increase specialization if species must specialize to avoid overlap. Further, pyrodiversity, again via species richness, may support interaction flexibility—enabling populations to resist perturbations.

In this study, I test for the first time whether environmental heterogeneity can influence a network's ability to resist severe environmental perturbations. Specifically, I test whether high pyrodiversity promotes resistance of a plant–pollinator network to drought, utilizing a natural fire history gradient in the Illilouette Creek Basin of Yosemite National Park, California. I focus on plant–pollinator communities because pollination interactions are ubiquitous across terrestrial systems (Ollerton, Winfree, & Tarrant, 2011), and severe droughts are known to affect both plants and pollinators (Alarcón, Waser, & Ollerton, 2008; Minckley et al., 2013). Previous work in this system found that higher pyrodiversity is associated with increased species richness of pollinators and flowering plants (Ponisio, Wilkin, et al., 2016). Here, I test whether pyrodiversity: (a) enhances functional redundancy, generalization, and/or complementarity of plant–pollinator networks, and (b) buffers populations against the decline of interaction partners by promoting partner flexibility. Lastly, I test whether pyrodiversity's effect on interaction patterns (c) increases community resistance to co‐extinction cascades. I expect that pyrodiversity will enhance population and community resistance to drought, especially for species that are flexible in their partners and network niche. This study is a unique empirical test of the relationship between ecological network structure and resistance to climate‐driven extremes.

## 2 METHODS

### 2.1 Study sites and collection methods

The study landscape was located in the Illilouette Creek Basin of Yosemite National Park, in the central Sierra Nevada of California. The basin is approximately 20,000 hectares and has never been logged or grazed at a commercial scale. The forest is upper elevation mixed‐coniferous, dominated by Jeffrey pine (*Pinus jeffreyi*), white fir (*Abies concolor*), red fir (*Abies magnifica*), and lodgepole pine (*Pinus contorta* var. *murrayana*). The forest is interspersed with meadows and shrublands. Fire was suppressed from the late 1800s until the early 1970s, when Yosemite National Park adopted a “let burn” management strategy that allows lightning‐ignited fires to run their course. After only approx. 30 years, the presuppression fire regime has been nearly restored, creating a burn patchwork of varying severity and age (Collins & Stephens, 2007). This system thus uniquely enables studies of pyrodiversity and its effects (Collins & Stephens, 2007).

**FIGURE 2:**
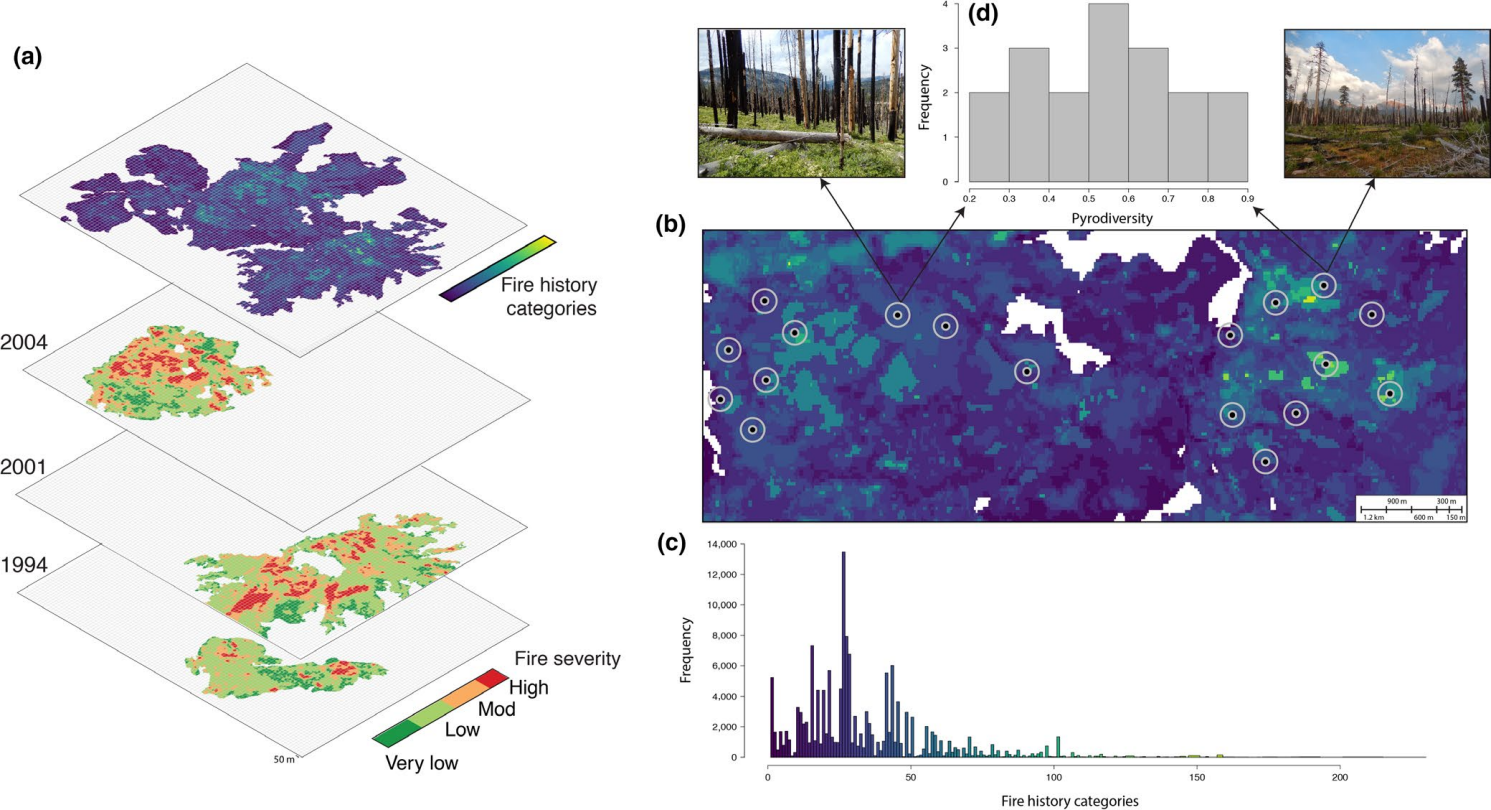
Representation of the method used to estimate pyrodiversity. (a) Each raster cell is assigned a fire history category based on the fires it experienced (three representative fires depicted). Areas that did not burn during the time period for which there is burn history data (i.e., wet meadows, riparian areas, large rocky outcrops, and fires that occurred before 1984; raster cells are white) are assigned their own fire history category. (b) The diversity of fire histories (pyrodiversity) is calculated within 150 m buffers (gray circles) around the monitoring sites (black point with gray outline).(c) The frequency of different unique fire histories within raster cells (135 categories) across the basin. (d) The frequency of the pyrodiversity scores calculated using Simpson's diversity of fire history categories within a buffer around a site. Representative sites are depicted for the highest and lowest pyrodiversity scores

I selected sites in order to cover a gradient of pyrodiversity across the basin. To estimate pyrodiversity, I used a metric to quantify fire history diversity in relation to the frequency, age, extent, and severity experienced in an area (Ponisio, Wilkin, et al., 2016). I first obtained fire history spatial data of the study area (spanning the entire basin) dating back to 1984 from Yosemite National Park and the United States Forest Service (Miller & Safford, 2012; van Wagtendonk et al., 2012; Yosemite National Park, 2012). Each fire‐specific spatial layer contains rasterized values of burn severity, classified according to the Relative difference Normalized Burn Ratio (RdNBR, Miller & Thode, 2007) at a 30 m^2^ resolution (Figure 2a). To estimate pyrodiversity, I evaluated the uniqueness of the fire history of each raster cell. I first created categories of fire severity within a fire (Miller & Safford, 2012). For each raster cell, I then used the sequence of fires and the severity of each of those fires to define unique fire histories. I identified 135 fire histories in the basin that were unique in some aspect (fire frequency, severity, and/or timing) from 1984 to when the sites were surveyed. For example, two raster cells were assigned different fire history categories if they burned in all the same fires, but at different severities (Figure 2b,c). I characterized the pyrodiversity surrounding a monitoring plot using Simpson's diversity index, treating fire history categories as “species.” Next, to calculate the abundance of each fire history category for the diversity index, I summed the number of cells of each unique fire history within a 150 m buffer centered on the plot (Figure 2b,d; Ponisio, Wilkin, et al., 2016). This buffer size was found to be most predictive of plant and pollinator richness in this study system (Ponisio, Wilkin, et al., 2016).

In 2013, with a team of field technicians, we established eighteen 50‐m^2^ monitoring sites across a gradient of pyrodiversity ranging from 0.2 to 0.9 (Simpson's diversity index) within the 150 m buffer (Figure 2d). The average distance between sites was 4 km, with a minimum of 500 m (greater than the foraging distance of most bees; Gathmann & Tscharntke, 2002). Sites span most of the basin and occur between 1,500 and 2,000 m above sea level; to ensure safety while sampling, sites were chosen within 2 km of a trail. We sampled pollinator communities June–August in 2013 and 2014. Each year, sites were sampled four times. Because sites were located 5–20 km from the nearest road, we accessed sites by backpacking and camping for 5‐ to 12‐day sampling rounds. In each round, sampling order was randomized. We conducted surveys under sunny conditions when the temperature was above 12°C and wind speed was below 2.5 m/s. We netted flower visitors for 1.5 hr of active search time (the timer was paused while handling specimens). All insect visitors that touched the reproductive parts of the flower were collected; however, this study focuses only on bees, the most abundant and efficient pollinators in the system. Bee specimens were identified to species (or morphospecies for the genera *Hylaeus*, *Nomada*, *Perdita*, *Sphecodes*, and *Lasioglossum* subgenus *Dialictus*) by expert taxonomists. Floral resources were also surveyed each time pollinators were sampled by recording each blooming plant species (excluding graminoids) and the approximate number of blooms in the monitoring site.

**FIGURE A1:**
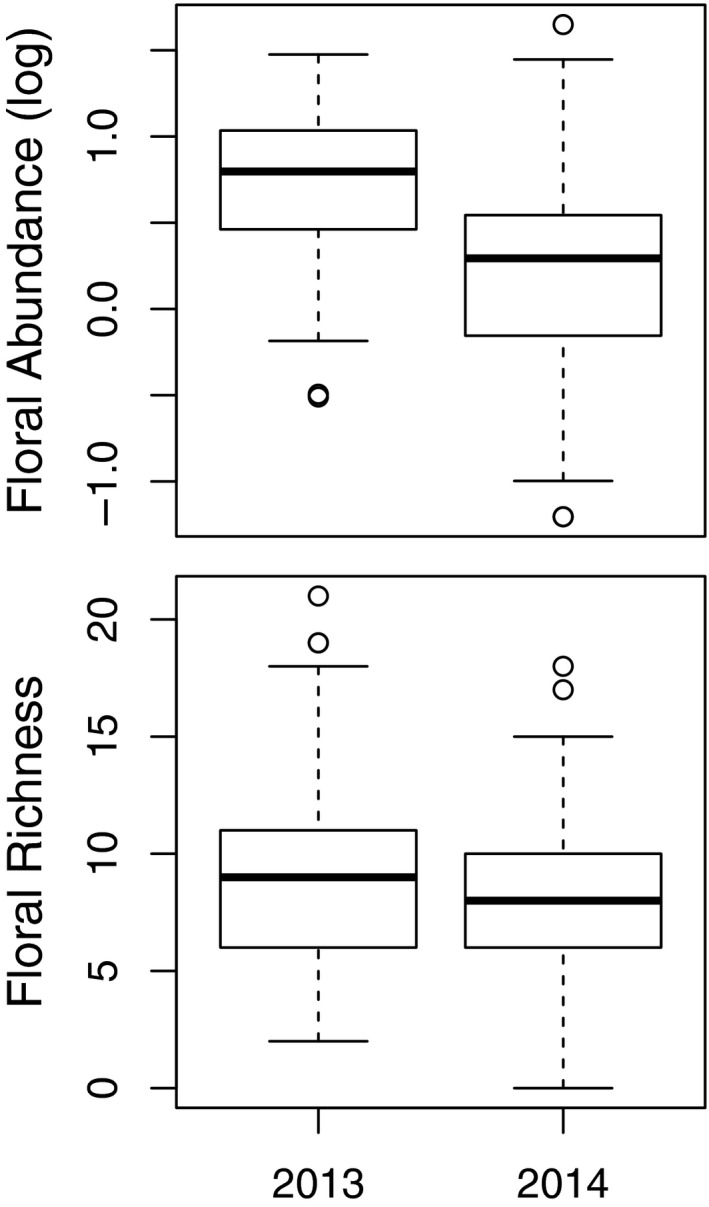
The log‐abundance and richness of flower plants between 2013 (severe drought) and 2014 (extreme drought). The abundance of flowers, but not richness was significantly lower in 2014. Blooms but not species were thus lost following the shift in drought intensity. Figure modified from Ponisio, Wilkin, et al. (2016)

During the study's collection period, the Sierra Nevada was experiencing a drought that began in 2012 (Griffin & Anchukaitis, 2014; Swain et al., 2014). In 2013, the intensity of the drought in the study area was categorized as “severe” (Griffin & Anchukaitis, 2014; Swain et al., 2014). In 2014, the drought conditions were upgraded to “extreme” and “exceptional”—the highest moisture deficit accumulation of any recorded span of previous years (Griffin & Anchukaitis, 2014; Swain et al., 2014). In the field, the impact of the extreme drought in 2014 was clear; stream and river water levels were lower throughout the season, and there was effectively no summer rainfall. In addition, many perennial plants such as *Ceanothus cordulatus* experienced dieback from exposure to freezing temperatures due to low snowpack levels. The abundance of blooms was lower in 2014 than in 2013 (Figure A1, Ponisio, Wilkin, et al., 2016). However, floral richness was not significantly different between years (Figure A1, Ponisio, Wilkin, et al., 2016), implying that communities lost individuals and blooms but not species.

### 2.2 Community resistance

#### 2.2.1 Network redundancy, complementarity, and generalization

To characterize the interaction network structure along the pyrodiversity gradient, I built interaction networks for each site and survey date, weighing interactions by their occurrence frequency. I calculated the functional redundancy of plant and pollinator interactions using Rao's metric (Bello, Lepš, Lavorel, & Moretti, 2007; Ricotta et al., 2016). Rao's metric estimates functional redundancy as the difference between species diversity (Simpson's) and Rao's functional trait diversity (Bello et al., 2007; Rao, 1982; Ricotta et al., 2016). Intuitively, if species diversity and functional trait diversity are the same value, then species are fully complementary and there is no redundancy (Rao's metric = 0). The converse is that if species diversity is much greater than functional diversity, functional redundancy is high (Rao's metric approaches 1). To calculate this metric, I used the interaction network as a trait matrix, where the “traits” of plants are the visiting pollinators, and vice versa from the pollinator's perspective. Therefore, plant functional redundancy is overlap in the use of bee species as pollinators. Similarly, pollinator functional redundancy is the overlap in plant visitation between bee species.

Functional complementarity of plants and pollinators was measured by constructing a dendrogram based on the differences in interaction partners between species of the same trophic level (using the bipartite function grouplevel; Devoto et al., 2012; Petchey & Gaston, 2007; Dormann, Frueund, Bluethgen, & Gruber, 2009). The branch lengths between species that overlap in partners will be shorter than the distance between species with few partners in common. Functional complementarity is then measured as the total branch lengths between species of the same trophic level. Lastly, to quantify plant and pollinator generalization, I calculated the mean number of partners per species for each trophic level (i.e., mean degree).

To test whether pyrodiversity affected network redundancy, generalization and complementarity, I included these network metrics as the response variables in linear mixed models with pyrodiversity as an explanatory variable (Bates, Mächler, Bolker, & Walker, 2015; Kuznetsova, Brockhoff, & Christensen, 2017). Because changes in partner availability have the potential to change interaction patterns, I included the interaction between pyrodiversity and drought intensity (severe/extreme). I included a random effect of site to account for the multiple surveys of each site. In order to determine whether richness was the mechanism underlying any responses to pyrodiversity, I also regressed network redundancy, generalization, and complementarity against species richness. I included a random effect of site in these models as well. All continuous explanatory variables were centered. In this and all subsequent models, I also used standard model assessment techniques to ensure that the assumptions of the models were met, and variance inflation factors (VIF) to estimate the collinearity between explanatory variables (Zuur, Ieno, & Elphick, 2010). Analyses were conducted in R 3.6.1 (R Core Team, 2019). The fully reproducible code and explanations for all analyses are available on GitHub at https://github.com/lponisio/Yosemite, https://doi.org/10.5281/zenodo.3647623.

#### 2.2.2 Co‐extinction cascade resistance

**FIGURE 3:**
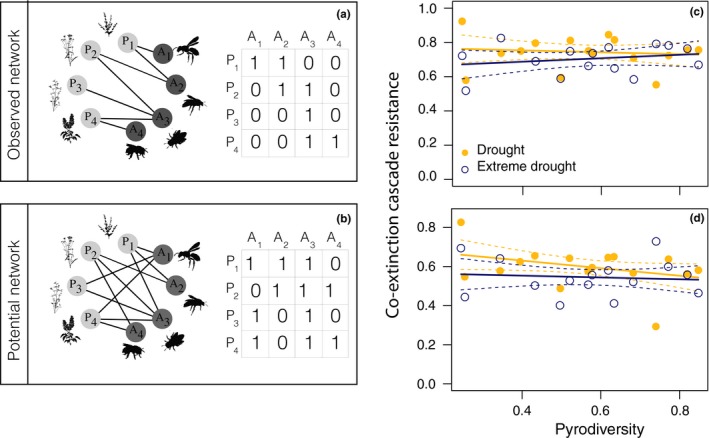
(a,b) Illustrates a hypothetical observed and potential interaction network. In the potential network, species that have ever been observed interacting are linked. (c,d) Pyrodiversity did not significantly affect the resistance to co‐extinction cascades of the potential networks before and after the extreme drought perturbation. Observed and potential networks showed qualitatively similar patterns. Plant species were removed sequentially by their abundance before drought. Points represent the average for a survey site. The solid line indicates the mean slope estimate, and the dashed lines are the 95% CI around the estimate. Slopes were not significantly different from zero

To quantify community resistance to extreme climatic events, I tested whether pyrodiversity lowers the probability of pollinator co‐extinction cascades (when an extinction of one species causes a partner's extinction) by simulating plant extinction. I considered two representations of the plant–pollinator networks at a monitoring site: (a) the traditional interaction matrix where an observed interaction between a plant and a pollinator is represented by a 1 in the corresponding cell (the “Observed network”; Figure 3a), and (b) if a pollinator was ever observed interacting with a certain plant species in any survey in the landscape, the hypothetical interaction was represented by a 1 in the corresponding cell (the “Potential network”; Figure 3b). The “Observed network” represents the realized interaction niche breadth of species, while the “Potential network” accounts for the possibility that species can re‐wire their interactions—approaching the fundamental partner niche breadth (Kaiser‐Bunbury et al., 2010). Next, for both network representations, I simulated plant species extinction and the subsequent co‐extinction of pollinator species (Memmott et al., 2004). I eliminated plant species based on abundance levels before the extreme drought—from lowest to highest—and then calculated the number of pollinators that subsequently went extinct. The assumption underlying this extinction simulation is that the least abundant species are most likely to be lost following a drought or other environmental perturbation (e.g., Tilman & El Haddi, 1992). I then used the simulated extinctions to generate a curve representing proportion of pollinator species remaining after the extinction of plant species, and used area below the extinction curve as an estimate of network resistance to co‐extinction cascades (i.e., network robustness, building on the bipartite function second.extinct; Memmott et al., 2004; Burgos et al., 2007; Dormann et al., 2009). When the area under the curve is equal to 1 (the maximum), this corresponds to a curve that decreases slowly until the point at which almost all plant species are eliminated. When the area is near zero (the minimum), this corresponds to when pollinators are lost abruptly after the loss of a single plant. To test whether pyrodiversity contributed to pollinator community resistance, I regressed community resistance against pyrodiversity. Like with the models of network metrics, I included an interaction between pyrodiversity and drought severity, and included site as a random effect (Bates et al., 2015; Kuznetsova et al., 2017).

#### 2.2.3 Population resistance

To evaluate pollinator resistance to shifts in the abundance of their floral partners, I quantified a population's ability to remain essentially unchanged as drought intensity increases. For each species present at a site in both years, I calculated the log‐ratio of abundance in 2014 (extreme drought) and 2013 (severe drought).

Next, I qualified a pollinator species' ability to adapt to changing conditions through flexibility in interaction partners and/or niche—a pollinators' placement within the network beyond its direct floral partners. To estimate a pollinator's ability to change floral interaction partners, I calculated interaction β‐diversity as pollinator partner variation within a year across sites and surveys (Anderson et al., 2011; Ponisio et al., 2017; Ponisio, M'Gonigle, & Kremen, 2016; Ponisio, Wilkin, et al., 2016). If a pollinator consistently interacted with the same plants at different sites across the landscape, the interaction β‐diversity value will be small.

I then quantified pollinator network niches and their ability to shift between those roles. I first selected a variety of metrics to characterize a pollinators' network role: (a) betweenness centrality, or how often the focal pollinator is present in the shortest path linking all species pairs in the network (González, Dalsgaard, & Olesen, 2010), (b) closeness centrality, or the average path length between the focal pollinator species and all other species in the network (González et al., 2010), (c) rarefied degree, or an approximate number of plant species a given pollinator species would have been observed visiting given more sampling—estimated using Chao2 (Chao, Colwell, Lin, & Gotelli, 2009; Ponisio et al., 2017; Winfree, Williams, Dushoff, & Kremen, 2014), (d) interaction niche overlap, measured using the mean Chao similarity index of interaction partners between the focal species and all other pollinators (Chao, Chazdon, Colwell, & Shen, 2005), (e) specialization, measured as the reciprocal specialization between a species pair (Blüthgen's d', Blüthgen, Menzel, & Blüthgen, 2006), and (f) plant dependence, or the sum of plant dependencies on a specific pollinator species (Bascompte, Jordano, & Olesen, 2006). I calculated indices using the specieslevel and networklevel functions within the R package bipartite (Dormann, 2011; Dormann et al., 2009).

To account for correlation between metrics, I combined the calculated species‐level network metrics into a single descriptor of a pollinator's network niche using principal component analysis (PCA) (Vidal et al., 2014). For each year, I first centered each metric across species and sites, and then used principal axis loading (PC1) to represent each pollinators' network niche for each year. To estimate pollinator network niche variability, I calculated the coefficient of variation (CV) of PC1 scores across the landscape in 2013 (pre‐extreme drought perturbation)—if a pollinator consistently occupies the same network niche at different sites across the landscape, the CV of the network niche PC1 score will be small. I also calculated the mean PC1 score to represent a species' average network niche (Vidal et al., 2014).

To determine what landscape or species characteristics influenced population resistance, I regressed population resistance (as measured by the log‐ratio of abundance) against (a) pyrodiversity, to test whether it increases community resistance, (b) both partner and niche variability, to determine whether a species' ability to be flexible in interactions contributes to its resistance, (c) the average network niche, to test whether a species' network niche is related to its population resistance, and (d) the mean log‐ratio of floral abundance (extreme/severe drought) at a site, as pollinator abundances are often tied to floral partner availability. To determine whether pyrodiversity fosters the conditions that allow flexible species to shift their partners and network niches, I also included an interaction between pyrodiversity and both metrics of interaction flexibility. Pollinator species identity was included as a random effect.

## 3 RESULTS

The survey team hand‐netted a total of 5,879 bee specimens comprising 143 species or morphospecies across 30 genera. Pollinator visitation was observed on 67 different flowering plant species and 793 unique plant–pollinator interactions. The most species‐rich bee genera were *Osmia* (34 species), *Andrena* (16 species), *Megachile* (12 species), and *Lasioglossum* (10 species/morphospecies). All plant and pollinator species were native except the European honey bee, *Apis mellifera*, which was common throughout the basin. Around 800 (15%) of the collected specimens were honey bees. In a 4‐year survey across all of Yosemite National Park, T. Griswold and colleagues collected around 520 bee species and morphospecies (T. Griswold, unpublished data), and our team collected 30% of those species in the 2‐year survey of the Illilouette Creek Basin alone.

### 3.1 Community resistance

#### 3.1.1 Network redundancy, complementarity, and generalization

**FIGURE 4:**
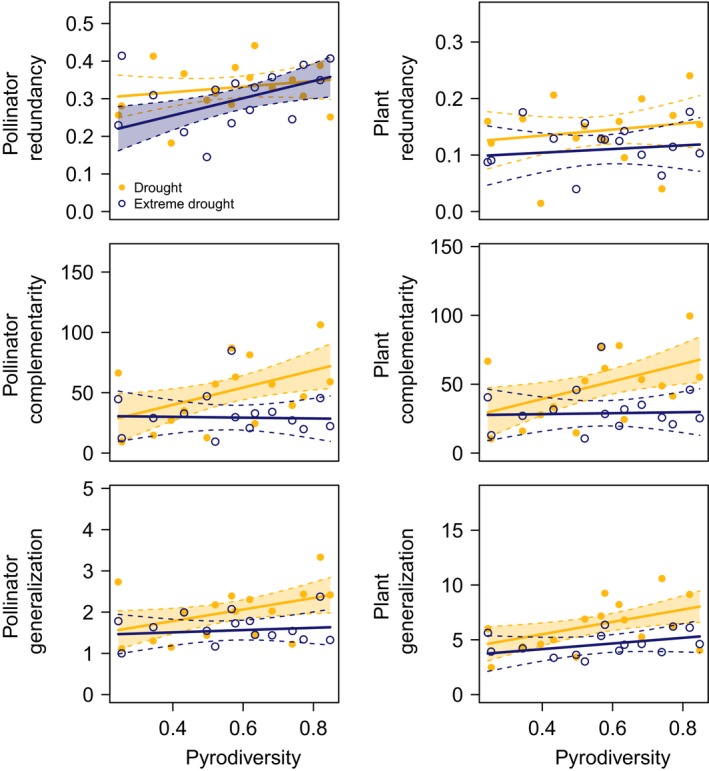
The relationships between pyrodiversity and plant functional redundancy (overlap in pollinator use by plants), pollinator functional redundancy (overlap in plant use by pollinators), plant functional complementarity (partitioning of pollinator use by plants), pollinator functional complementarity (partitioning of plant use by pollinators), and plant/pollinator generalization (average number of unique partners) before and after the extreme drought perturbation. Points represent the average for a survey site. The solid line indicates the mean slope estimate, and the dashed lines are the 95% confidence intervals (CI) around the estimate. CI are filled with color when the slope is significantly different from zero

Pyrodiversity was not consistently significantly related to plant or pollinator functional redundancy (Figure 4, Table 1), but was significantly positively related to functional complementarity of both trophic levels (before the extreme drought; Figure 4, Table 1). Plant generalization and pollinator generalization were also significantly positively related to pyrodiversity before the extreme drought (Figure 4, Table 1). This translates to an average of one extra plant partner for bees, and four additional pollinator species visiting per day for plants in the most pyrodiverse sites.

**TABLE 1:** The estimates (± *SE*), test statistics, degrees of freedom (*df*), and *p*‐values for the linear mixed models of network metrics and pyrodiversity or species richness

Network metrics	Pyrodiversity			Richness		
	Estimate ± *SE*	*t*‐value*_df_*	*p*‐value	Coefficient ± *SE*	*t*‐value*_df_*	*p*‐value
Pollinators						
Redundancy	0.013 ± 0.014	0.95_15.13_	N.S.	0.02 ± 0.005	3.54_362.1_	.0005***
Δ drought	−0.034 ± 0.007	−5.18_386.59_	3.60e^(−07)^			
Complementarity	12.66 ± 4.87	22.60_17.17_	0.019	12.91 ± 1.91	6.76_354.38_	5.80e^(−11)^***
Δ drought	−22.94 ± 2.77	−8.27_386.58_	2.13e^(−15)^			
Generalization	0.25 ± 0.11	2.2_16.17_	0.043	0.32 ± 0.045	7.12_129.33_	6.43e^(−11)^***
Δ drought	−0.47 ± 0.059	−8.02_387.63_	1.25e^(−14)^			
Plants						
Redundancy	0.009 ± 0.012	0.77_14.84_	N.S.	0.024 ± 0.005	4.75_160.3_	N.S.
Δ drought	−0.035 ± 0.008	−4.32_387.12_	2.02e^(−05)^			
Complementarity	11.24 ± 4.36	2.58_17.45_	0.019	12.26 ± 2.06	5.95_154.05_	1.76e^(−08)^***
Δ drought	−21.50 ± 2.62	−8.21_388.90_	3.37e^(−15)^			
Generalization	1.00 ± 0.38	2.64_17.82_	0.017	1.08 ± 0.16	6.83_279.75_	5.39e^(−11)^***
Δ drought	−1.89 ± 0.24	−7.90_389.27_	2.82e^(−14)^			

Note

For pyrodiversity, the change in intercept (i.e., the change in the mean because the explanatory variables are scaled) between the drought and extreme drought years (Δ drought) is also reported. *, **, and *** indicate significance at the .05, .01, and .001 levels, respectively.

**FIGURE 5:**
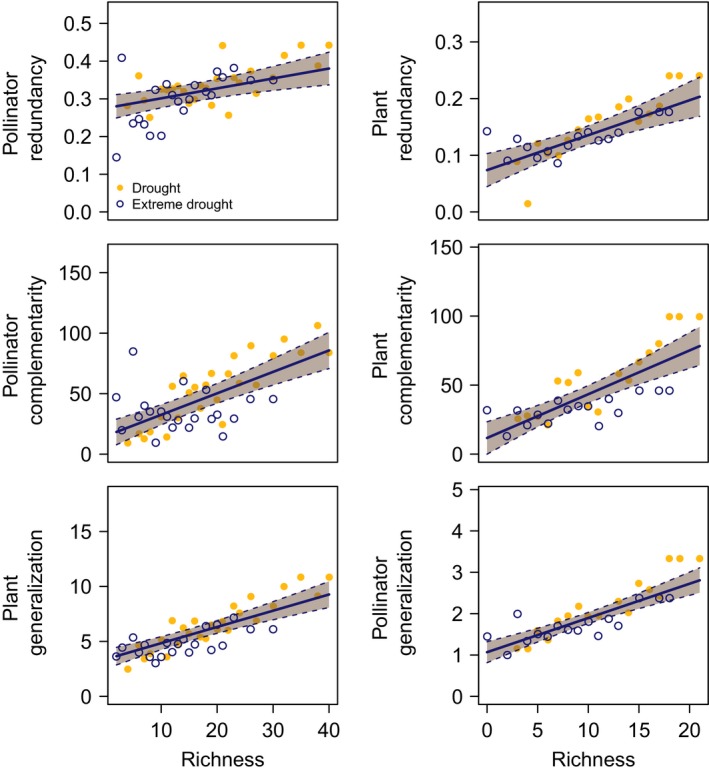
The relationships between species richness and plant/pollinator functional redundancy, functional complementarity, and generalization. Pollinator metrics are regressed against pollinator species richness, except in the case of pollinator generalization, which is regressed against plant species richness. Similarly, plant metrics are regressed against plant species richness, except in the case of plant generalization, which is regressed against pollinator species richness. Note that the bottom panels are switched relative to Figure 3 in the main text to align *x*‐axes. Points represent the average for a survey site. The solid line indicates the mean slope estimate, and the dashed lines are the 95% confidence intervals (CI) around the estimate. CI are filled with color when the slope is significantly different from zero

Plant and pollinator functional redundancy, complementarity, and generalization were all significantly lower in the extreme drought year (Figure 4, Table 1). Plants interacted with an average of two fewer pollinator species, and pollinators interacted with one fewer plant species (Figure 4, Table 1). Only the redundancy of plant use by pollinators was significantly positively related to pyrodiversity in the extreme drought year (interaction between pyrodiversity and the extreme drought ± *SE*, 0.027 ± 0.008, *t*‐value = 3.38_387.80_, *p*‐value = .0008). All the network metrics examined were significantly positively related to species richness (Figure 5, Table 1). All model VIF were <2 (Zuur et al., 2010).

#### 3.1.2 Co‐extinction cascade resistance

Co‐extinction cascade resistance was not significantly related to pyrodiversity (Figure 3c,d), both with and without interaction re‐wiring. The extreme drought significantly lowered co‐extinction cascade resistance (Figure 3, estimate of the difference in mean resistance between years ± *SE* of the estimate, −0.05 ± 0.013, *t*‐value = −3.96_389.88_, *p*‐value = 9.08e−05). Pyrodiversity interacted significantly with drought severity, indicating that sites with high pyrodiversity maintained community resistance more in the extreme drought year than did sites with lower pyrodiversity (estimate of interaction between pyrodiversity and drought intensity ± *SE* of the estimate, 0.026 ± 0.013, *t*‐value = 1.99_390.70_, *p*‐value = .047). When all possible interaction partners were represented in the network, the results of the linear model did not change qualitatively (Figure 3c vs. d). All VIF were <2 (Zuur et al., 2010).

#### 3.1.3 Population resistance

**FIGURE 6:**
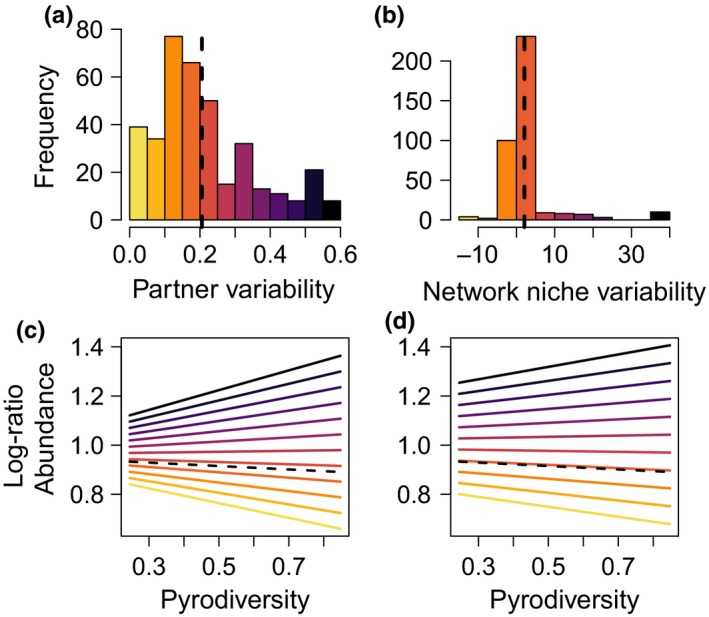
Pyrodiversity and partner/network niche variability interact to determine species resistance to drought‐mediated changes in plant abundance. The top histograms (a,b) depict the distribution of partner and network niche variability in the pollinator community. The mean is indicated by a dashed line. The effect of pyrodiversity on the log‐ratio of pollinator abundance depends on the pollinator's partner and network niche variability (c,d). Different levels of partner and niche variability are represented by colors, matched between the histogram and line graphs

**FIGURE A2:**
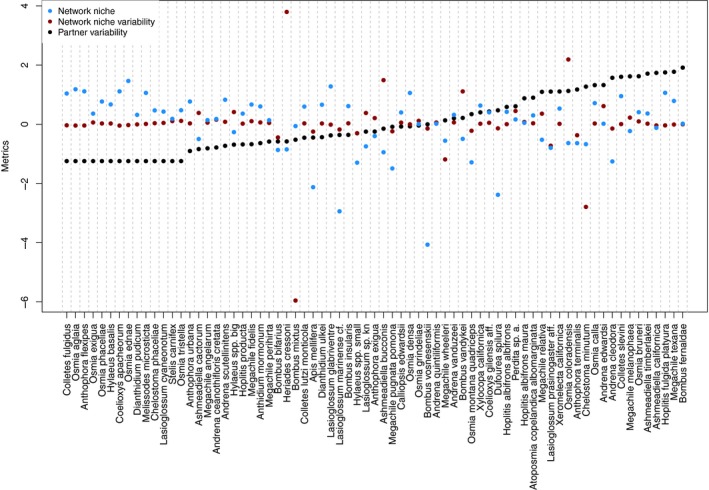
The average network niche (blue), network niche variability (red), and partner variability (black) of bee species. Species are sorted from highest to lowest partner variability. Metrics are standardized so the mean is a 0

**FIGURE A3:**
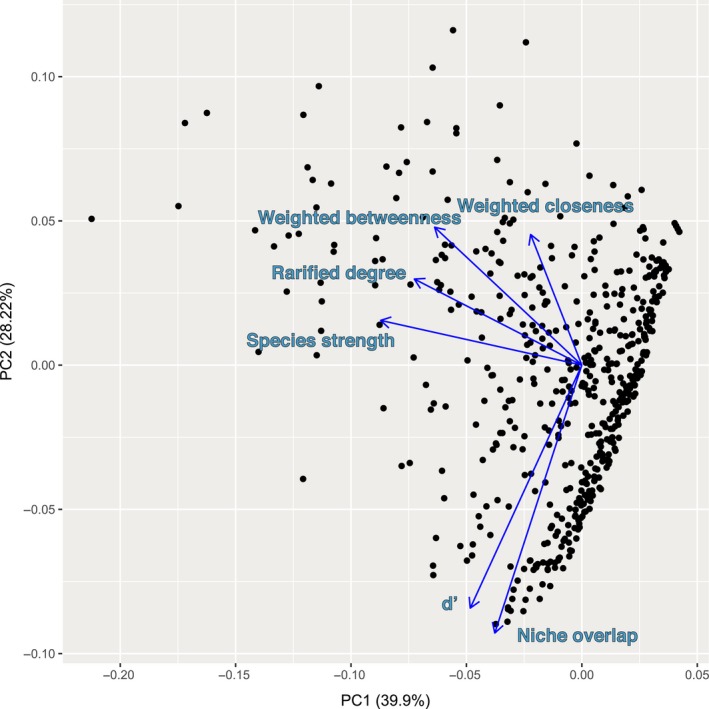
The PCA of network roles. Each point represents a species' network role at a specific site and survey

Species varied in both their partner (Figures 6a, A2) and network niche variability (Figures 6a, A2). The PC1 axis, describing a species network role, explained 40% of the variance (Figure A3). The PC1 loadings for each of the network niche metrics were, in descending order, plant dependence (−0.60), rarefied degree (−0.50), betweenness centrality (−0.44), reciprocal specialization (−0.33), interaction niche overlap (−0.26), and closeness centrality (−0.15).

Pyrodiversity interacted with both metrics of interaction flexibility to significantly influence population‐level resistance to drought (Figure 6, estimate of the interaction between partner variability and pyrodiversity ± *SE* of the estimate, 0.188 ± 0.041, *t*‐value = 4.57_171.07_, *p*‐value = 9.22e−06; network niche variability and pyrodiversity 0.075 ± 0.030, *t*‐value = 2.44_147.17_, *p*‐value = .016). Pollinators with above‐average partner and network niche flexibility at sites with high pyrodiversity maintained their population or increased in abundance postextreme drought (log‐ratio abundance > =1; Figure 6c,d). Pollinators with average interaction flexibility tended to have smaller populations after the drought perturbation (log‐ratio < 1) and did not respond to pyrodiversity (Figure 6c,d). Pollinators with below‐average interaction flexibility declined strongly after the drought perturbation (log‐ratio < 1) and responded negatively to pyrodiversity. In addition, the average network niche of a species was significantly related to species resistance to drought (estimate of the slope of network niche and resistance ± *SE* of the estimate, 0.109 ± 0.044, *t*‐value = 2.50_155.74_, *p*‐value = .013). The change in floral abundance at a site did not have a significant effect on species resistance. All VIF were <2 (Zuur et al., 2010).

## 4 DISCUSSION

Pyrodiversity had surprising effects on the plant–pollinator network and its response to an extreme drought event. Pyrodiversity promoted functional complementarity via its effects on species richness, suggesting more pyrodiverse areas will support more total pollination function (Blüthgen & Klein, 2011; Devoto et al., 2012). Pyrodiversity also facilitated interaction flexibility at the population level, enabling species to respond to changes in community composition. Species that can shift partners and network niches are better able to take advantage of the heterogeneity generated by pyrodiversity, thereby buffering pollinator populations against changes in their partners' abundances. However, because pyrodiversity did not enhance functional redundancy or resistance to co‐extinction cascades, it is unlikely to improve community‐level resistance to droughts. This suggests that even the landscape heterogeneity generated by pyrodiversity has limited potential to buffer communities from extreme changes in interaction partner richness.

### 4.1 Community resistance

Before the extreme drought, pyrodiversity promoted plant and pollinator functional complementarity as well as interaction generalization. Though functional complementarity and generalization are commonly increased at the expense of each other, here pyrodiversity increased species richness enough for plant and pollinator species to both partition their partners while also expanding the number of species with which they interact. Bees interacted with an average of two plant species per day and gain an extra partner in the most pyrodiverse areas. Given this is a 50% increase in a bee's diet breadth, this increase is likely biologically significant. Similarly, plants have an average of 6.5 bee species visiting per day and gain an additional four partners in the most pyrodiverse areas—a substantial increase in their potential pollinators. Interestingly, the combination of increased pollinator complementarity (reducing resistance) and generalization (increasing resistance) is likely why pyrodiversity did not significantly affect co‐extinction cascade resistance.

During the extreme drought, pyrodiversity was no longer significantly related to plant/pollinator interaction complementarity or generalization. With fewer interaction partners to choose from, pollinators visit fewer plants with more partner overlap. More overlap in partners translated to increased pollinator interaction redundancy at the most pyrodiverse sites, though the average functional redundancy of both plants and pollinators across the landscape decreased significantly in the extreme drought. In addition, while the increased redundancy interactions in the most pyrodiverse areas may provide some resistance to future disturbance, here the interaction reorganization appears to be a product of pollinators visiting the only plants available, which may intensify interspecific competition. These findings suggest that the drought‐mediated changes in species richness cascade through the community, affecting interaction patterns and likely lowering the total community function. This study provides further support that extreme climatic events are a significant threat to terrestrial biodiversity (Sheffield & Wood, 2008).

### 4.2 Population resistance

At the population level, pyrodiversity interacted with species interaction flexibility, shaping the resistance of pollinator populations to drought. Specifically, species that can shift partners and network niches are better able to take advantage of the heterogeneity generated by pyrodiversity—buffering pollinator populations against changes in plant/partner abundances. Given the greater floral diversity in pyrodiverse areas (Ponisio, Wilkin, et al., 2016), flexible pollinator species will often change their partners and network niche (Cuartas‐Hernández & Medel, 2015; Gómez & Zamora, 2006; MacLeod, Genung, Ascher, & Winfree, 2016; Ponisio et al., 2017; Spiesman & Gratton, 2016; Waser, Chittka, Price, Williams, & Ollerton, 1996). The negative response of the least flexible species to pyrodiversity may be because pyrodiverse sites were able to support pollinators with specific plant preferences before the extreme drought, but not after. Although species‐level patterns of pollen resource use tend to be phylogenetically conserved (Minckley & Roulston, 2006), it is unclear whether interaction flexibility is a phylogenetically conserved trait (MacLeod et al., 2016). Further exploration of the ecological, behavioral, and physiological mechanisms that enable interaction flexibility is crucial if we aim to better predict species success in new and changing environments. Interestingly, the bee species that was most able to change its partners and network niche—thereby increasing in average abundance during the extreme drought—was *A. mellifera*, an introduced species in California. Its high partner and network flexibility may be related to *A. mellifera*'s successful global invasion and its dominance as a floral visitor in the communities it has invaded (Hung, Kingston, Albrecht, Holway, & Kohn, 2018).

The ability of a pollinator population to survive drought‐meditated declines in floral resource abundance was also significantly related to its average network niche. Lower values of the network niche metric were related to higher centrality (betweenness, closeness, degree), plant dependence, reciprocal specialization, and interaction niche overlap (Figure A3); species that occupy these network niches were more likely to lose individuals following the increase in drought intensity. From the plant's perspective, interaction strength is positively correlated with pollinator interaction frequency (Vázquez et al., 2012), and centrality within plant–pollinator network is positively related to plant fitness (Gómez & Perfectti, 2012). In addition, Brosi and Briggs (2013) found that interaction specialization positively impacts the reproduction of *Delphinium barbeyi*, a perennial, pollinator‐dependent forb. It follows then that the pollinator species that decline most in abundance during the drought perturbation (high centrality, reciprocal specialization, and plant dependence) are likely the most important for maintaining pollination services. Though the measure of importance to network structure varies, Vidal et al. (2014) also found that in plant–frugivore networks, the animals that contribute most to the network are the most vulnerable. Simulated co‐extinction cascades suggest plant–pollinator communities are relatively resistant to the loss of species, except when species that are important to maintaining the network are lost first (Memmott et al., 2004). If this positive relationship between a species' function and its vulnerability is more general, previous studies (Kaiser‐Bunbury et al., 2010; Memmott et al., 2004) may overestimate community resistance.

### 4.3 Limitations

**FIGURE A4:**
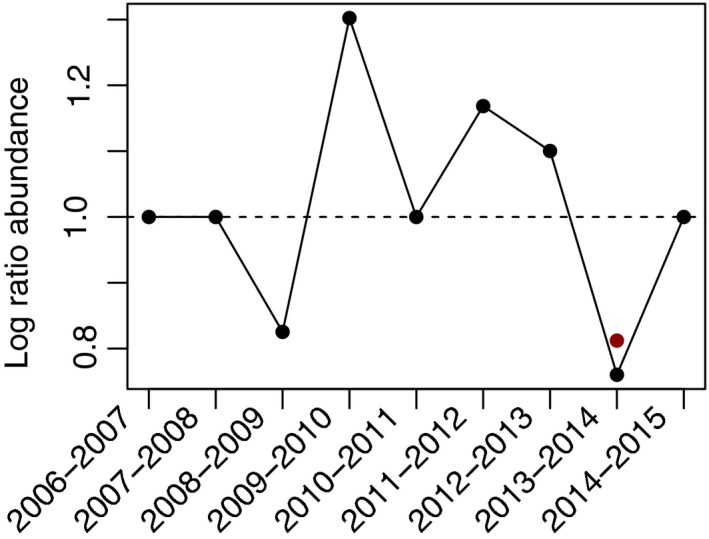
The average change in abundance of species (log‐ratio) between years in a long‐term survey of bee communities in Northern California (Kremen et al., 2018; Ponisio Gaiarsa & Kremen, 2017; Ponisio, M'Gonigle, et al., 2016) (black points) and this study (red point). When the log‐ratio is 1 (dashed line), populations maintain their abundance between years. The Northern California study has approximately the same sampling design as this study, but sites were only sampled for 1 hr of active searching (vs. 1.5 hr in this study)

A limitation of this study is that it examines a single shift in drought intensity. The difficult and time‐consuming nature of community‐level sampling and species identification prevented replication of this study until the drought ended three years later in 2017. However, though pollinator communities are naturally temporally variable (e.g., Alarcón et al., 2008; Petanidou, Kallimanis, Tzanopoulos, Sgardelis, & Pantis, 2008; Ponisio et al., 2017), in comparison with a longer‐term Northern California community‐level dataset collected using similar methods from 2006 to 2015 (Kremen, M'Gonigle, & Ponisio, 2018; Ponisio et al., 2017; Ponisio, M'Gonigle, et al., 2016), it is clear the population changes observed in this study are more extreme than in other years (Figure A4). In the longer study, the average log‐ratio of abundance between sequential pairs of years was near 1 (populations stayed relatively constant in size) in four of the nine years sampled (Figure A4). In a comparison of the log‐ratio of abundance in different pairs of years in the Northern California sites, the 2013–2014 is the only year significantly lower than the other years (Table A1). This suggests the largest decrease in population sizes was observed in 2013–2014 when the shift in drought intensity took place across California, and therefore, this study captures the appropriate pair of years to investigate the effect of an extreme drought perturbation.

## 5 CONCLUSIONS

This study contributes to our understanding of the factors that affect the provision of ecosystem function through species interactions. Pyrodiverse landscapes promote functional complementarity and generalization and—while pyrodiversity does not consistently enhance community‐level functional redundancy—it does support flexible behaviors that enable species to resist perturbations at the population level (Oliver et al., 2015). Factors associated with climate change and land management such as fire suppression are eroding pyrodiversity by promoting homogeneous “megafires” (Dellasala, Williams, Williams, & Franklin, 2004; Moritz et al., 2012; Noss, Franklin, Baker, Schoennagel, & Moyle, 2006; Stephens et al., 2014) instead of historically patchy, mixed‐severity fires. This study suggests that predicted shifts toward less‐diverse fire regimes will negatively influence population resistance and ecosystem function in this and other forested ecosystems. Wildland Fire Use programs, such as those implemented in the Illilouette Basin, can restore fire regimes, and this study finds further support that they are integral for promoting biodiversity through pyrodiversity (Ponisio, Wilkin, et al., 2016; Van Wagtendonk, 2007).

## CONFLICT OF INTEREST

The authors declare no conflict of interest.

## AUTHOR CONTRIBUTION

**Lauren C. Ponisio:** Conceptualization (equal); data curation (equal); formal analysis (equal); funding acquisition (equal); investigation (equal); methodology (equal); project administration (equal); resources (equal); validation (equal); visualization (equal); writing—original draft (equal); and writing—review and editing (equal).

### Open Research Badges

This article has been awarded Open Data and Open Materials Badges. All materials and data are publicly accessible via the Open Science Framework at https://github.com/lponisio/Yosemite, and Zenodo https://doi.org/10.5281/zenodo.3647623

## Data Availability

Data are deposited in GitHub with the analysis code (https://github.com/lponisio/Yosemite, and Zenodo (https://doi.org/10.5281/zenodo.3647623), as well as in Dryad.

## APPENDIX A

**TABLE A1:** The estimates (±*SE*), test statistics, degrees of freedom (*df*), and *p*‐values for the linear mixed models of abundance log‐ratios in Northern California plant–pollinator communities

Year pair	Estimate ± *SE*	*t*‐value*_df_*	*p*‐value
2006–2007 (Intercept)	1.31 ± 0.14	9.49_577_	<2e−16***
2007–2008	−0.19 ± 0.17	−1.11_577_	N.S.
2008–2009	−0.30 ± 0.17	−1.76_577_	N.S.
2009–2010	0.16 ± 0.22	0.75_577_	N.S.
2010–2011	−0.31 ± 0.23	−1.34_577_	N.S.
2011–2012	0.18 ± 0.17	1.05_577_	N.S.
2012–2013	−0.02 ± 0.15	−0.16_577_	N.S.
2013–2014	−0.43 ± 0.15	−2.91_577_	.004**
2014–2015	−0.03 ± 0.16	−0.16_577_	N.S.

Note

*, **, and *** indicate significance at the .05, .01, and .001 levels, respectively. Only the 2013–2014 year pair has log‐ratios that are significantly lower than the other comparisons.
